# The double-pass correlation: A reliability metric for subjective judgments in developmental research

**DOI:** 10.1016/j.mex.2026.104039

**Published:** 2026-07-09

**Authors:** Ryoichi Watanabe, Yusuke Moriguchi

**Affiliations:** a Research Organization of Open Innovation and Collaboration, Ritsumeikan University, 2-150 Iwakura-cho, Ibaraki, Osaka, 567-8570, Japan; b Graduate School of Letters, Kyoto University, Yoshidahonmachi, Sakyo Ward, Kyoto, 606-8501, Japan

**Keywords:** Children, Reliability, Double-pass-correlation, Similarity-judgement

## Abstract

Ensuring reliable psychological experiments with young children is challenging, especially when outcomes depend on subjective judgments that vary across individuals and tasks. The double-pass design, in which identical trials are presented twice to assess within-participant consistency, is useful for evaluating reliability. However, the number of repeated trials required for reliable estimation in developmental samples remains unclear. We reanalyzed the data from 30 children aged 3–6 years and 31 adults who completed two blocks of color pair similarity judgments. In the observed order analysis, adults reached stable correlations with approximately 31 trials under a strict criterion and 14 trials under a lenient criterion, whereas children required approximately 50 and 32 trials, respectively. Children reached ρ = .30 after approximately 49 repetitions, whereas stronger thresholds were less consistent: 83.3% exceeded ρ = .50 and 63.3% exceeded ρ = .70 in the full 81-trial estimates. These findings suggest that approximately 50 repeated trials may be appropriate for young children when feasible, whereas at least 32 repeated trials may be needed as a minimum shortened procedure to obtain reliable results. For adults, approximately 31 trials may suffice. These results provide empirical guidance for designing shorter, developmentally appropriate tasks that maintain reliable interpretability.

## Abstract (author highlights)

• Reanalyze an existing developmental dataset, including children and adults.
• Identify how reliability changes as repeated judgments accumulate.
• Provide practical benchmarks for designing shorter double-pass tasks for young children.

## Graphical abstract

**Figure:**
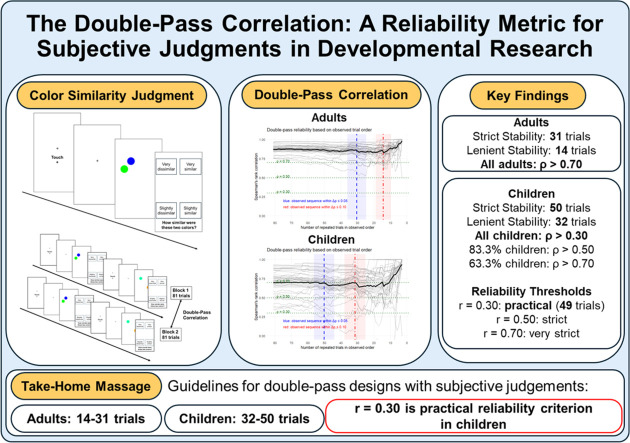

## Specifications table

**Table:** 

**Subject area**	Psychology
**More specific subject area**	Developmental Psychology
**Name of your method**	Double-Pass Correlation
**Name and reference of original method**	
**Resource availability**	

## Background

Reliability is crucial for interpreting behavioral data in developmental populations, where attention, motivation, task understanding, and response consistency can cause noise [1]. In developmental psychology, this issue has been examined across a range of relatively objective paradigms, including categorization tasks, reaction-time tasks, looking-time measures, accuracy-based designs, or psychophysical procedures [2,3]. Researchers have developed and evaluated these objective measurements such as quality using test–retest reliability [4], internal consistency [5], performance-based exclusion criteria [6], and trial number optimization [7]. Thus, reliability is a central methodological concern that is assessed using explicit criteria in many behavioral paradigms [8].

Subjective judgment measures are widely used in developmental research, especially in metacognition and conscious experience studies [6,[9], [10], [11], [12]]. However, establishing their reliability is methodologically challenging. Unlike objective indices, subjective judgments lack verifiable correct answers and rely on unstable decision-making. Variability in responses may reflect individual differences or inconsistencies, complicating the quantification of reliability, determination of required observations, and acceptable consistency levels.

A promising method for assessing within-individual reliability is the double-pass design, in which some trials are repeated to examine the response consistency to identical stimuli [13,14]. Introduced in psychophysics, the double-pass method provides a direct index of perceptual and decisional noise by comparing responses across repeated presentations [[15], [16], [17]]. Extensions into higher-level cognitive tasks, such as lexical decision, show that double-pass measures capture reliability across many domains [18].

Moriguchi et al. (2025) used a double-pass framework to assess the reliability of children’s and adults’ subjective color similarity judgments [9]. Participants rated the perceptual similarity between the two color stimuli on a four-point scale. Repeating color pairs quantified judgment consistency while examining developmental differences. The results showed age-related improvements in double-pass correlations, identifying r = .30 as a criterion for adequate reliability. The findings highlighted the difficulty of full double-pass protocols in younger children: among 73 children aged 3–4 years, 13 failed to complete the task, 14 had the double-pass correlations below .30, and four further exclusions were based on other criteria, totaling 31 exclusions. Among the 162 children aged 5–6 years, one failed to complete the task, 13 had the double-pass correlations below .30, and four were excluded based on other criteria, totaling 18 exclusions. By contrast, exclusion rates were low among children aged 7 years and older and adults. Thus, younger children showed signs of fatigue and reduced feasibility in the full study design.

Despite growing interest, two questions remain. First, the number of repeated trials required for a stable double-pass correlation in young children is unclear. Second, because repeating all trials is impractical, it is crucial to determine whether partial double-pass designs with repeated trials can reliably estimate the threshold. Guidelines would help researchers balance data quality with practical constraints, improving subjective judgment tasks in developmental research.

This study reanalyzed datasets to determine the repeated trials needed for stable double-pass correlations in young children. Using cumulative correlations from various repeated trials, we identified when estimates stabilized and examined reliability thresholds in subjective judgment research (ρ = 0.30, 0.50, and 0.70). Our goal is to guide the implementation of partial double-pass designs that minimize task demands while maintaining reliable measurements. These findings contribute to the development of robust, child-friendly methods for assessing subjective judgments in developmental research.

## Method details

### Participant

Experiment 4 of Moriguchi et al. (2025) originally included 41 children aged 3–6 years [mean age = 66.56 months, SD = 11.11; 21 girls] and 31 adults [mean age = 22.19 years, SD = 2.31; 14 women]. The experiment was conducted in person in a laboratory using a visual similarity judgment task. Of the original sample, 11 children were excluded due to prespecified technical problems or exclusionary criteria.

The present reanalysis included 30 children and 31 adults who completed the task and fulfilled the inclusion criteria. All participants completed the tasks individually during a laboratory session. Children were tested in a child-friendly room in the presence of their parents (s), whereas adults were tested independently. The study protocol was approved by the Ethics Committee of the Psychological Science Unit, Kyoto University (No. 3-P-22).

### Apparatus and testing environment

The task was administered on an iPhone 15 Plus with a 6.7-inch Super Retina XDR OLED display (2796 × 1290 pixels, 460 ppi). The experiment was performed in a room with controlled ambient illumination provided by standard ceiling-mounted lighting without direct sunlight. The display was calibrated before the experiment to ensure a consistent color presentation across participants.

### Stimuli and task

**Fig. 1:**
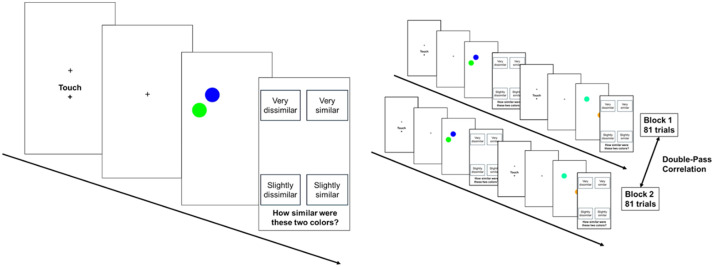
The color similarity judgement task and double-pass correlation setting.

*Note*. Figures are adapted from Moriguchi et al. (2025).

Details of the task are shown in Fig. 1. The task included all 81 possible unordered pairs of nine colors (blue, light blue, aquamarine, green, light green, orange, red, pink, and purple), following the specifications of the original study [[19], [20], [21]]. In each trial, two colored circles were presented at different locations on the screen. Participants rated the similarity of the pair on a four-point scale (1 = “very dissimilar,” 2 = “slightly dissimilar,” 3 = “slightly similar,” 4 = “very similar”). The 81 trials were presented in a random order for each participant’s judgment.

The participants completed two blocks of 81 trials. Block 2 repeated the identical stimulus pairs from Block 1, enabling the computation of the double-pass consistency.

Our analysis focused on estimating the minimum number of repeated trials required to obtain a stable and practically interpretable double-pass correlation. Because the task used a four-point ordinal rating scale, we computed Spearman’s rank correlations between the responses in Blocks 1 and 2.

We first conducted an observed order analysis based on the original trial sequence. For each participant and trial number N, Spearman’s rank correlation was calculated using the first N repeated trial pairs in the actual order in which they were administered. This analysis preserved the temporal structure of the task and allowed us to examine how double-pass reliability changed over the course of the experiment, including the possible effects of practice, fatigue, or changes in attention.

We then conducted a random sampling analysis to examine the effect of the number of repeated trials independently of the original trial order. For each participant and each trial number N, we randomly sampled N repeated trial pairs without replacement from the full set of 81 repeated pairs and calculated Spearman’s rank correlation between the first and second presentations of sampled pairs. This procedure was repeated 1000 times for each value of N, yielding a distribution of reliability estimates for each trial number. The mean correlation across iterations was used to describe the participant-level reliability trajectory ρ(N) as a function of the number of repeated trials.

To evaluate convergence, we compared the correlation obtained at each trial number N with the full double-pass correlation obtained from all 81 repeated trials ρ(81). In the observed order analysis, this comparison was based on the correlation estimated from the first N trials in the original sequence. In the random sampling analysis, a comparison was conducted for each random sample at each value of N. Because we aimed to identify the point at which the estimate became practically stable rather than testing the null hypothesis, we adopted a tolerance-based criterion. Together, these analyses allowed us to evaluate double-pass reliability in the actual temporal context of the task and in a manner independent of the original trial order.

Group-level means and 95% confidence intervals were calculated across participants. Confidence intervals were computed for the mean number of required trials using the mean ± t₀.₉₇₅, df × SD / √n, where SD is the standard deviation of the participant-level estimates, n is the number of participants, and df = n − 1.

### Stability

Specifically, for each participant and trial number N, we calculated the absolute deviation between the partial trial estimate and the full 81 trial estimate, |ρ(N) − ρ(81)|. In the observed order analysis, ρ(N) was calculated from the first N repeated pair trials in the original sequence. In the random sampling analysis, ρᵢ(N) was calculated from the i th random sample of N repeated trials, where i denotes the sampling iteration.

For the observed order analysis, a trial number was considered stable when the partial trial estimate fell within the prespecified margin relative to the full 81 trial estimate. For the random sampling analysis, a trial number was considered stable when at least 95% of the 1000 random sampling estimates fell within the same margin. We examined both a strict criterion, |ρ(N) − ρ(81)| ≤ 0.05, and a more lenient criterion, |ρ(N) − ρ(81)| ≤ 0.10. In the random sampling analysis, these criteria were applied to the iteration-specific estimates |ρᵢ(N) − ρ(81)|. These margins were treated as practical tolerances, indicating that the partial estimate was sufficiently close to the full estimate for a substantive interpretation.

### Reliability

In addition to the convergence analyses, we examined the number of repeated trials required for the cumulative double-pass correlation to exceed the predefined reliability thresholds of ρ = 0.30, 0.50, and 0.70. These thresholds were used as practical benchmarks corresponding to moderate, strong, and very strong correlation-based reliability.

For the observed order analysis, we identified the smallest number of repeated trials for each participant at which the cumulative Spearman correlation, ρ(N), calculated from the first N trials in the original sequence, exceeded each threshold. For the random sampling analysis, we applied the same threshold analysis to the participant-level reliability trajectory, defined as the mean Spearman correlation across 1000 sampling iterations at each trial number N.

## Method validation

**Fig. 2:**
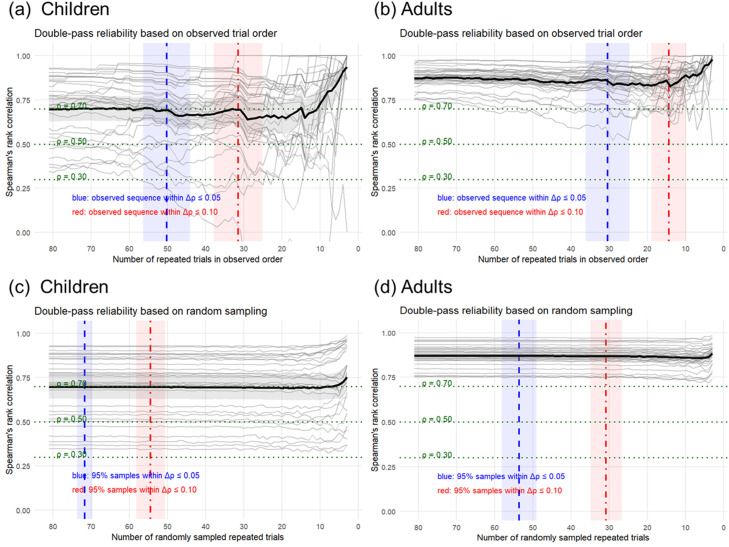
Double-pass correlations for children (left panels) and adults (right panels).

*Note*. The y-axis shows Spearman’s rank correlation between the two repeated blocks, and the x-axis indicates the number of repeated trials included in the partial correlation ρ(N). Moving rightward corresponds to fewer repeated trials. Panels (a) and (b) show the observed order analysis based on the original trial sequence for children and adults, respectively. Panels (c) and (d) show the random sampling analysis for children and adults, respectively. The thin gray lines represent the individual participants. The thick black line shows the mean across participants, and the gray ribbon denotes the 95% CI. The blue dashed line indicates the strict stability criterion (|Δρ| ≤ 0.05), and the red dot-dashed line indicates the lenient criterion (|Δρ| ≤ 0.10). The green dotted lines indicate, from bottom to top, the moderate (ρ =.30), strong (ρ =.50), and very strong (ρ =.70) criteria.

Fig. 2 illustrates the progression of the partial double-pass correlations as a function of the number of repeated trials.

## Stability

### Observed order analysis

The observed order cumulative correlation analysis revealed the number of repeated trials required for Spearman double-pass correlations to reach stability under two absolute deviation criteria: a strict criterion, |Δρ| ≤ 0.05, and a more lenient criterion, |Δρ| ≤ 0.10, relative to the full 81 trial estimate.

For children, the mean double-pass correlation based on all 81 repeated trials was ρ = .698 (95% CI [.629, .766]). Partial correlations stabilized after an average of 50.2 trials (95% CI [44.0, 56.3]) under the stricter criterion, whereas the lenient criterion indicated stability after 31.5 trials (95% CI [25.1, 37.8]) (Fig. 2a). At 50 repeated trials, one child fell below the reliability cutoff of ρ = .30. At 32 repeated trials, three children fell below the cutoff. Nevertheless, at these corresponding trial numbers, the mean correlations remained close to the full trial estimate: ρ = .692 (95% CI [.622, .763]) at 50 trials and ρ = .695 (95% CI [.615, .775]) at 32 trials.

For adults, the mean double-pass correlation based on all 81 repeated trials was high (ρ =.871, 95% CI [.849,.893]). Partial correlations stabilized after an average of 30.5 trials (95% CI [24.7, 36.2]) under the stricter criterion, whereas the lenient criterion indicated stability after 14.4 trials (95% CI [9.81, 19.0]) (Fig. 2b). No adult fell below the reliability cutoff of ρ = .30 under both criteria. The mean correlations remained high, although slightly lower than the full trial estimate: ρ = .864 (95% CI [.832, .896]) at 31 trials and ρ = .828 (95% CI [.789, .867]) at 14 trials.

We then examined whether the stability criteria derived from one age group could be applied to the other. When the adult-derived trial numbers were applied to the child data, three children fell below the reliability cutoff of ρ = .30 under both criteria. The mean correlations remained well above this cutoff: ρ = .696 (95% CI [.611, .780]) at 31 trials and ρ = .649 (95% CI [.553, .746]) at 14 trials.

Conversely, when the trial numbers derived from the child data were applied to the adult data, all adults exceeded the reliability cutoff of ρ = .30 under both criteria. The mean correlations remained high and were virtually identical to the full trial estimate: ρ = .865 (95% CI [.839, .892]) at 50 trials and ρ = .859 (95% CI [.828, .891]) at 32 trials.

Taken together, the observed order analysis suggests that the full 81 repeated trials may not always be necessary to obtain stable and practically interpretable double-pass correlation values. However, the number of repeated trials required for stable reliability estimates differed across age groups. Children required more repeated trials than adults, and applying adult-derived trial numbers to the child data led to some children falling below the practical reliability cutoff of ρ = .30. In contrast, child-derived trial numbers provided highly reliable estimates for adults. These results indicate that shortened double-pass procedures can reduce the task burden; however, age-appropriate trial numbers are needed, particularly when assessing subjective judgments in children.

### Random sampling analysis

The random sampling cumulative correlation analysis further examined whether the estimated number of repeated trials was robust when the original trial order was maintained. As in the observed order analysis, stability was evaluated under two absolute deviation criteria: a strict criterion, |Δρ| ≤ 0.05, and a more lenient criterion, |Δρ| ≤ 0.10, relative to the full trial estimate of 81.

For children, partial correlations stabilized after an average of 71.7 trials (95% CI [69.7, 73.7]) under the stricter criterion, whereas the lenient criterion indicated stability after 54.4 trials (95% CI [50.7, 58.2]) (Fig. 2c). No child fell below the reliability cutoff of ρ = .30 for either criterion. At these corresponding trial numbers, the mean correlations were virtually identical to the full trial estimate, with ρ = .697 (95% CI [.629, .765]) at both 72 and 54 trials.

For adults, partial correlations stabilized after an average of 53.6 trials (95% CI [49.0, 58.2]) under the stricter criterion, whereas the lenient criterion indicated stability after 30.8 trials (95% CI [26.7, 34.9]) (Fig. 2d). No adult fell below the reliability cutoff of ρ = .30 for either criterion. At these corresponding trial numbers, the mean correlations remained virtually unchanged from the full trial estimate: ρ = .870 (95% CI [.848, .892]) at 54 trials and ρ = .869 (95% CI [.847, .890]) at 31 trials.

We then examined whether the stability criteria derived from one age group could be applied to the other. When the adult-derived trial numbers were applied to the child data, all children exceeded the reliability cutoff of ρ = .30 under both criteria. The mean correlations remained well above this cutoff: ρ = .697 (95% CI [.629, .765]) at 54 trials, and ρ = .695 (95% CI [.627, .762]) at 31 trials.

Conversely, when the trial numbers derived from the child data were applied to the adult data, all adults exceeded the reliability cutoff of ρ = .30 under both criteria. The mean correlations remained high and were virtually identical to the full trial estimate: ρ = .871 (95% CI [.849, .892]) at 72 trials, and ρ = .870 (95% CI [.848, .892]) at 54 trials.

Together, the random sampling analysis confirmed that the double-pass procedure could be shortened without substantially compromising the reliability estimation when the trial order was not preserved. However, children require more repeated trials than adults to achieve stable reliability estimates, indicating that age-specific trial lengths may be necessary.

Overall, the observed order and the random sampling analyses suggest that 81 repeated trials may not always be necessary to obtain stable and practically interpretable double-pass correlation values. Because the observed order analysis preserves the original temporal structure of the task, it provides a direct and practical estimate of the number of repeated trials required in the actual experimental context. For children, approximately 50 repeated trials under the strict criterion and 32 trials under the lenient criterion produced correlations close to the full 81 trial estimate. For adults, approximately 31 and 14 repeated trials were sufficient under strict and lenient criteria, respectively.

## Reliability

### Observed order analysis

To determine the number of repeated trials required to exceed practical reliability thresholds, we examined the observed order Spearman correlation, ρ(N), across different numbers of repeated trials. In this analysis, ρ(N) was calculated using the first N repeated pairs in the original trial sequence.

All children exceeded the moderate threshold of ρ = .30 in all 81 trial estimates (Fig. 2a). Reaching this threshold required an average of 49.3 trials (95% CI [31.7, 67.0]). Reaching ρ = .50 required an average of 59.9 trials (95% CI [42.5, 77.3]). Although not all children reached this threshold, 83.3% (25/30) exceeded ρ = .50 in the full 81 trial estimate. For the very strong threshold of ρ = .70, the mean required trial count was 69.6 trials (95% CI [61.7, 77.6]), with 63.3% (19/30) of the participants achieving this level in the full 81 trial estimate.

In contrast, all adults ultimately exceeded all three thresholds (ρ =.30,.50, and.70) in the full 81 trial estimate (Fig. 2b). For the ρ = .30 and ρ = .50 thresholds, adults remained above the cutoff across all examined trials. Reaching ρ = .70 required an average of 35.8 trials (95% CI [20.5, 51.0]).

Taken together, these findings suggest that approximately 49 repeated trials may be needed for children to reliably reach a moderate double-pass reliability criterion (ρ = .30), whereas substantially fewer trials may be sufficient for adults. Stronger criteria, such as ρ = .50 and ρ = .70, may be informative, but they appear overly stringent for some young children in this task.

### Random sampling analysis

To determine whether the reliability threshold results were robust when the original trial order was not preserved, we examined the random sampling based Spearman correlation ρ(N) across different numbers of repeated trials.

All children exceeded the moderate threshold of ρ = .30 in all 81 trial estimates (Fig. 2c). Reaching ρ = .50 required an average of 68.3 trials (95% CI [46.1, 90.4]). Although not all children reached this threshold, 83.3% (25/30) exceeded ρ = .50 in the full 81 trial estimate. For the very strong threshold of ρ = .70, the mean required trial count was 65.2 trials (95% CI [49.7, 80.7]) with 63.3% (19/30) of the participants achieving this level in the 81 trial estimate.

In contrast, all adults ultimately exceeded all three thresholds (ρ =.30,.50, and.70) in the full 81 trial estimate (Fig. 2d).

Taken together, the observed order and random sampling analyses showed that children required more repeated trials than adults to obtain reliable double-pass correlations. Children required approximately 49 trials to reach the moderate reliability threshold of ρ = .30, and stronger thresholds were reached less consistently: 83.3% of children exceeded ρ = .50, and 63.3% exceeded ρ = .70 in the full 81 trial estimate. In contrast, adults exceeded all three thresholds in all 81 trial estimates.

## Conclusion

The present findings suggest that the number of repeated trials in a shortened double-pass procedure should be selected according to age group and the intended level of reliability. Based on the observed order analysis, approximately 50 repeated trials may be recommended as a conservatively shortened procedure for young children. This number corresponds to the strict stability criterion and produces correlations close to the full trial estimate of 81. For adults, approximately 31 repeated trials may be sufficient as a conservatively shortened procedure.

When reducing participant burden is especially important, approximately 32 repeated trials may be considered a lower-bound option for children. This value was close to both the lenient stability estimate and the lower bound of the 95% confidence interval for the number of trials required to reach ρ = .30. However, because three children fell below the practical reliability cutoff at 32 trials, this shorter version should be used cautiously. Thus, for young children, approximately 50 repeated trials are recommended when feasible, whereas at least approximately 32 trials should be retained when the task burden must be minimized.

## Limitations

The proposed method has several limitations. First, the validation data was obtained from a narrow age range and a specific visual color similarity judgment task. Reliability trajectories may differ in younger or older children and in tasks involving other perceptual domains or different response formats.

Second, the practical cutoffs and stability criteria used in this study should not be regarded as universal standard. Although they provide useful empirical benchmarks, the optimal number of repeated trials may vary depending on task demands, participant characteristics, and the level of reliability required for a specific research question. Accordingly, the present benchmarks should be interpreted as task-specific guidance rather than fixed criteria for all developmental studies.

Third, although the observed order and random sampling analyses provide useful complementary estimates, they cannot fully capture all the factors that may influence children’s performance during an actual experiment, including fatigue, practice, motivation, and attention fluctuations. Future studies should examine whether the present benchmarks can be generalized to other tasks, age groups, and experimental contexts.

## Ethics statements

The study protocol was approved by the Ethics Committee of the Psychological Science Unit, Kyoto University (No. 3-P-22).

## Supplementary material *and/or* additional information [OPTIONAL]

The code and data of the analysis of the study are available here (https://osf.io/9fw3u).

## Declaration of generative AI and AI-assisted technologies in the manuscript preparation process

During the preparation of this work the authors used ChatGPT and Paperpal in order to do English-language editing. After using this tool/service, the authors reviewed and edited the content as needed and take full responsibility for the content of the published article.

## CRediT authorship contribution statement

**Ryoichi Watanabe:** Conceptualization, Formal analysis, Methodology, Visualization, Writing – original draft. **Yusuke Moriguchi:** Conceptualization, Methodology, Writing – review & editing.

## Declaration of competing interest

The authors declare that they have no known competing financial interests or personal relationships that could have appeared to influence the work reported in this paper.

## Data Availability

The code and data of the analysis of the study are available (https://osf.io/9fw3u).

## References

[1] Witton C., Talcott J.B., Henning G.B. (2017). Psychophysical measurements in children: challenges, pitfalls, and considerations. PeerJ.

[2] Zelazo P.D., Lourenco S.F., Frank M.C., Elison J.T., Heaton R.K., Wellman H.M. (2021). Measurement of cognition for the national children’s study. Front. Pediatr..

[3] Hessels R.S., Hooge I.T.C. (2019). Eye tracking in developmental cognitive neuroscience - The good, the bad and the ugly. Dev. Cogn. Neurosci..

[4] Beck D.M., Schaefer C., Pang K., Carlson S.M. (2011). Executive function in preschool children: test-retest reliability. J. Cogn. Dev..

[5] Williams A., Steele J.R. (2016). The reliability of child-friendly race-attitude implicit association tests. Front. Psychol..

[6] Watanabe R., Moriguchi Y. (2023). Young children’s subjective and objective thresholds and emergent processes of visual consciousness using a backward masking task. Conscious. Cogn..

[7] Arnon I. (2020). Do current statistical learning tasks capture stable individual differences in children? An investigation of task reliability across modality. Behav. Res. Methods.

[8] Zorowitz S., Niv Y. (2023). Improving the reliability of cognitive task measures: a narrative review. Biol. Psychiatry Cogn. Neurosci. NeuroimAging.

[9] Moriguchi Y., Watanabe R., Sakata C., Zeleznikow-Johnston A., Wang J., Saji N. (2025). Comparing color qualia structures through a similarity task in young children versus adults. Proc. Natl. Acad. Sci. USA.

[10] Watanabe R., Tsuchiya N., Qianchen L., Myowa M., Moriguchi Y. (2025). How much can children see and report about their experience of a brief glance at a natural scene?. Neurosci. Conscious..

[11] Watanabe R., Moriguchi Y. (2025). Thresholds and emergent processes of visual consciousness on color and category in preschoolers and adults. Sci. Rep..

[12] Watanabe R., Moriguchi Y. (2024). Development of emergent processes and threshold of consciousness with levels of processing. Front. Psychol..

[13] Burgess A.E., Colborne B. (1988). Visual signal detection. IV. Observer inconsistency. J. Opt. Soc. Am. A.

[14] Green D.M., Swets J.A. (1966). Signal detection theory and psychophysics.

[15] Awwad Shiekh Hasan B., Joosten E., Neri P. (2012). Estimation of internal noise using double passes: does it matter how the second pass is delivered?. Vision. Res..

[16] Neri P. (2010). Visual detection under uncertainty operates via an early static, not late dynamic, non-linearity. Front. Comput. Neurosci..

[17] Neri P. (2010). How inherently noisy is human sensory processing?. Psychon. Bull. Rev..

[18] Diependaele K., Brysbaert M., Neri P. (2012). How Noisy is Lexical Decision?. Front. Psychol..

[19] Kawakita G., Zeleznikow-Johnston A., Takeda K., Tsuchiya N., Oizumi M. (2025). Is my “red” your “red”?: evaluating structural correspondences between color similarity judgments using unsupervised alignment. iScience.

[20] Kawakita G., Zeleznikow-Johnston A., Tsuchiya N., Oizumi M. (2024). Gromov-Wasserstein unsupervised alignment reveals structural correspondences between the color similarity structures of humans and large language models. Sci. Rep..

[21] Zeleznikow-Johnston A., Aizawa Y., Yamada M., Tsuchiya N. (2023). Are color experiences the same across the visual field?. J. Cogn. Neurosci..

